# The influence of graded levels of *Cyathula prostrata* (Linn.) Blume on semen quality characteristics of adult New Zealand white bucks

**DOI:** 10.1093/tas/txaa060

**Published:** 2020-06-19

**Authors:** Peter Kelechi Ajuogu, Richard Ere, Medubari B Nodu, Chinwe Uchechi Nwachukwu, Osaro O Mgbere

**Affiliations:** 1 School of Science and Technology, University of New England, Armidale, New South Wales, Australia; 2 Department of Animal Science, Faculty of Agriculture, University of Port Harcourt, Rivers State, Nigeria; 3 Department of Animal Science and Fisheries, Faculty of Agriculture, Niger Delta University, Amasoma Bayelsa State, Nigeria; 4 Department of Agricultural Science, Alvan Ikoku Federal College of Education, Owerri, Imo State, Nigeria; 5 School of Veterinary Medicine and Science, University of Nottingham, Nottingham, UK; 6 Department of Pharmaceutical Health Outcomes and Policy, University of Houston College of Pharmacy, Texas Medical Center, Houston, TX

**Keywords:** *Cyathula prostrata* (Linn.) Blume, New Zealand white buck, semen profile

## Abstract

*Cyathula prostrata* (Linn.) Blume is a tropical herbal plant known for its important phytochemical contents and medicinal properties. But its impact on animal reproduction and fertility is yet to be fully established. Therefore, we tested the hypothesis that *C. prostrata* (Linn.) Blume will improve the semen quality characteristics of New Zealand White buck rabbit. Twenty-eight post-pubertal buck rabbits were used for the study. The animals were randomly assigned to four treatment groups (*n* = 7 per treatment) where they were fed either the control diet—0 g *C. prostrata* (Linn.) Blume or any of the three experimental diets containing the graded levels of *C. prostrata* (Linn.) Blume incorporated into rabbit pellets at 10, 20 or 30 g *C. prostrata* (Linn.) Blume per kg feed. The results showed that the semen volume and pH were not different between groups. Interestingly, sperm motility significantly decreased (*P* < 0.05) in a dose-dependent manner. Similarly, the sperm morphology also decreased in a dose-related fashion with 20 g (77.75 ± 1.31%) and 30 g (79.00 ± 2.20%) *C. prostrata* (Linn.) Blume being significantly (*P* < 0.05) lower compared with groups 0 g (88.50 ± 1.44%) and 10 g (87.50 ± 4.33%) *C. prostrata* (Linn.) Blume, respectively. In conclusion, the addition of *C. prostrata* (Linn.) Blume into the normal rabbit feeds had a positive effect on sperm count, but reduced sperm motility and morphology, and may be associated with spermatogenesis-related problems.

## INTRODUCTION

Herbal derived natural products have long been sources of medicine and form about 25% of pharmaceuticals in modern medicine (Pan et al., 2013). There is an overwhelming interest in the use of herbal drugs/phytopharmaceuticals to complement conventional drugs (Calixto et al., 1998), especially with the recent increase in conventional drug resistance (Ibekwe et al., 2000). Several herbal plants used in African traditional medicine have demonstrated male fertility enhancement or regulatory potentials such as increased sexual desire (Subramoniam et al., 1997; Zheng et al., 2000), improvement of hormonal stimulatory activity (Xu et al., 2003; Ajuogu et al., 2019), and increased semen quality characteristics (Gonzales et al., 2001; Nantia et al., 2009; Ajuogu et al., 2019). Some of the herbal plants are reported to produce antifertility effects, which include an impairment on sperm production capacity, reduced semen quality (Reddy et al., 1997; Shaik et al., 2017; Ajuogu et al., 2018), reduced serum testosterone and luteinizing hormone (LH) levels (Hadley et al., 1981), and disrupted the hypothalamic–pituitary axis (Hadley et al., 1981; Nair and Bhiwgade, 1990).

Semen is a mixture of spermatozoa, produced by testicles, and seminal fluids secreted by the accessories glands, which are combined at the time of ejaculation (Haschek et al., 2010). Its analysis provides measurement on the volume and appearance of the semen, sperm size and shape (morphology), sperm movement (motility), concentration (pH), and number of the spermatozoa per ejaculate. Importantly, semen and sperm evaluation is a crucial procedure in determining the fertility and reproductive health of the individual (Colenbrander et al., 2003; Petrunkina et al., 2007). Morphologically, the impairment of semen parameters, such as a decrease in sperm count and sperm motility, are indication of compromised fertility status (Vazquez-Levin et al., 1997; Redmon et al., 2002; Pasqualotto et al., 2005). Kuete (2014) reported that fertility in male animals may be hindered or affected by toxicity of chemical or phytochemical agents, leading to a reduction in sperm production by the testis, maturation of sperm in the epididymis, and decrease in quality of sperm characteristics available for fertilization. The study further stated that interference with hormonal control of spermatogenesis by disruption/imbalance occurring within hypothalamic–pituitary–testicular axis might affect gonadotropins, LH, and follicle-stimulating hormone (FSH) production. Furthermore, Tempest et al. (2008) recons that male infertility is largely due to defects of spermatogenesis evidenced through the production of low-quality sperm or compromised sperm with reduced ability to fertilize a female egg and promote healthy embryonic development. Buck rabbits are often used for such andrological studies due to ease in semen collection (Skinner, 1967; Carson and Amann, 1972).

*Cyathula prostrata* (Linn.) Blume is a tropical annual, slender herbaceous plant of the *amaranth* family, known for its medicinal properties and phytochemical content. It grows in cultivated areas, wasteland, and forest margins in tropical and subtropical environments and has important crude traditional therapeutic uses in many tropical and subtropical countries including Nigeria, Cameron, Ghana, Ivory Coast, China, and Australia (Burkill, 1985; Mbatchi et al., 2006; Zheng and Xing, 2009). It is one of the highly exploited tropical herbal plants, with important phytochemical content such as terpenoids, flavonols, phenols, and tannins, steroids and carbohydrates. Its medicinal properties include anti-inflammatory and analgesic properties (Ibrahim et al., 2012), antibacterial and antifungal activities (Oladimeji et al., 2005; Unni et al., 2009; Ogu et al., 2012), antidiabetes (Ogbonnia et al., 2016), and has been used to treat rheumatism, eye problems, dysentery, otitis, and wound healing (Burkill, 1985).

In addition, ethanolic extract of *C. prostrata* (Linn.) Blume has been reported as an effective agent against cervical and breast cancer cells (Sowemimo et al., 2009). In Eastern Côte d’Ivoire, *C. prostrata* (Linn.) Blume is used to maintain pregnancy in the last trimester and also help to ease labor and delivery during childbirth (Malan and Neuba, 2011). Despite these numerous medical properties, its link with animal reproduction biology has not been fully established. Since there is no known study that documents the effect of *C. prostrata* (Linn.) Blume on semen quality characteristics in rabbit bucks, we designed this study to investigate whether *C. prostrata* (Linn.) Blume will enhance or deteriorate the semen quality profile in adult New Zealand White buck rabbits.

## MATERIALS AND METHODS

### Ethical Approval

The research study was reviewed and approved by the Research and Ethics Committee of the Faculty of Agriculture Research and Ethics Committee, University of Port Harcourt, Rivers State, and conformed to the NIH standards of care for the use of experimental animals and good research practice.

### Location of the Experiment

The experiment was setup at the rabbitry unit of the Rivers State University of Science and Technology, Faculty of Agriculture, Teaching and Demonstration Farm, Port Harcourt, Nigeria. Port Harcourt is the capital and largest city in Rivers State, Niger Delta Region, Nigeria, and is located along Bonny River upstream from the Gulf of Guinea.

### Experimental Design and Management

Twenty-eight post-pubertal New Zealand White buck rabbits acquired from the University of Port Harcourt Teaching Research Farm, Port Harcourt, Nigeria, aged 7 to 8 mo with an average weight of 2.5 kg were used for the study. The animals were housed individually in a conventional single-tier rabbit hutches at the rabbitry unit of the Rivers State University of Science and Technology, Faculty of Agriculture Teaching and Demonstration Farm under standard conditions—12 h light, 12 h dark, 24 ± 2 °C temperature, 60 ± 3% humidity with free access to feed and water. After preconditioning them for 2 wk, they were randomly distributed equally (*n* = 7 bucks/group) into four treatment groups designated as 0 g (control), 10, 20, and 30 g *C. prostrata* (Linn.) Blume. The bucks in control group received feed free of *C. prostrata* (Linn.) Blume treatment (0 g/kg feed), while those in the treatment groups 10, 20, and 30 g *C. prostrata* (Linn.) Blume received feed mixed with 10, 20, and 30 g of *C. prostrata* (Linn.) Blume per kilogram feed.

### Experimental Diet Preparation

Fresh *C. prostrata* (Linn.) Blume plants (whole plant) were harvested at Orowurukwu Village, near the Rivers State University of Science and Technology, Orowurukwu, Port Harcourt, Nigeria. The plant was identified and authenticated by the Taxonomy Unit of Forestry and Wildlife Department at the University where the plant specimen was also deposited. *Cyathula prostrata* (Linn.) Blume (whole plant) were sun-dried and reduced to powder using a blending machine. Graded levels of *C. prostrata* (Linn.) Blume powder was administered into crumbled commercial rabbit pellets feed obtained from Top Feeds Nigeria Limited as follows: groups 0 g (control), 10, 20, and 30 g *C. prostrata* (Linn.) Blume per kg feed.

### Ejaculation Procedure and Collection of Samples

A reinforced polyvinyl chloride tube was assembled to form artificial vagina according to the methods described by Herbert and Adejumo (1996) and modified by Ajuogu et al. (2015). The bucks were sexually prepared by allowing three false mounts on the doe used as a teaser before collection to enhance quality (Macmillan and Hafs, 1967). Semen samples were collected from each rabbit in the treatment groups with the aid of artificial vagina (AV) as described by Herbert and Adejumo (1996). Before use, the AV device was placed in a warm bath (40 to 60 °C) for 10 to 15 min (Ajuogu et al., 2015). A mature nongravid doe was used as a teaser. As the buck mounts and makes a thrust on the teaser doe before intromission, the AV was applied from the rear and this elicits ejaculation within a few seconds.

The samples were collected twice from all the bucks between 9 and 10 am in weeks 7 and 8 of the experimental period. The ejaculate collected from the collecting tubule was read directly from the glass tubule to determine the semen volume. The semen quality characteristics were assessed by routine methods of microscopic examination. Semen pH was measured using pH-indicator strips; sperm motility, morphology, and sperm count per ejaculates were estimated according to the method described by Blom (1981). Semen volume (mL) is a measure of volume of semen collected in one ejaculation, sperm count measures the number of sperm per milliliter of semen in one ejaculation, sperm morphology (%) is the index used to measure percentage sperm that have a normal shape by evaluating the structure of 500 spermatozoa with normal morphological structure as classified by Blom (1981) and sperm motility (%) determines the percentage of sperm that have normal movement.

### Statistical Analysis

Data obtained from the treatments were subjected to one-way analysis of variance using completely randomized design as the model (*Y*_*ij*_ = μ + *T*_*i*_ + *e*_*ij*_). The treatment means were separated using Duncan’s new multiple range test. Data management and statistical analysis were conducted using Minitab Statistical Software (State College, PA).

## RESULTS

Table 1 shows the semen profile of the rabbit bucks fed graded levels of *C. prostrata* (Linn.) Blume. The total sperm count significantly increased (*P* < 0.05) with inclusion levels, resulting in the highest total sperm count in groups fed 10 g (1.6 ± 0.5 million/mL) and 20 g (1.5 ± 0.3 million/mL) *C. prostrata* (Linn.) Blume compared with the value in control group 0 g *C. prostrata* (Linn.) Blume (0.9 ± 0.2 million/mL). There was no effect due to treatment (*P* > 0.05) on semen volume and pH in groups 0 g (1.15 ± 0.002; 8.40 ± 0.14), 10 g (1.10 ± 0.002; 8.35 ± 0.12), 20 g (1.10 ± 0.003; 7.85 ± 0.18), and 30 g (1.20 ± 0.003; 8.40 ± 0.18) *C. prostrata* (Linn.) Blume. Also, the treatment effect on sperm motility showed a significant (*P* < 0.05) decrease in a dose-dependent manner. The groups fed 0 g (86.25 ± 2.39%) and 10 g (78.25 ± 4.50%) *C. prostrata* (Linn.) Blume were significantly better (*P* < 0.05) than groups fed 20 g (76.25 ± 3.12%) and 30 g (75.25 ± 0.25%) *C. prostrata* (Linn.) Blume. The morphology of the semen showed similar downward trend in a dose-related fashion in groups on 0 g (88.50 ± 1.44%) and 10 g (87.50 ± 4.33%) being significantly (*P* < 0.05) better than treatment groups 20 g (77.75±1.31%) and 30 g (79.00±2.2%) *C. prostrata* (Linn.) Blume. There were no significant (*P* > 0.05) treatment effects on the semen volume and pH.

**Table 1.** Effect of *Cyathula prostrata* (Linn.) Blume on semen Profile of rabbit bucks

Treatment group				
Parameters	0 g (control)	10 g	20 g	30 g
Semen volume, mL	1.15 ± 0.002	1.10 ± 0.002	1.10 ± 0.003	1.20 ± 0.003
pH	8.40 ± 0.14	8.35 ± 0.12	7.85 ± 0.18	8.40 ± 0.18
Motility, %	86.25 ± 2.39^b^	78.25 ± 4.50^b^	76.25 ± 3.12^a^	75.25 ± 0.25^a^
Morphology, %	88.50 ± 1.44^b^	87.50 ± 4.33^b^	77.75 ± 1.31^a^	79.00 ± 2.20^a^
Total sperm count, million/mL	0.90 ± 0.20^a^	1.60 ± 0.50^b^	1.50 ± 0.30^b^	1.10 ± 0.60^ab^

Treatment group: 0, 10, 20, and 30 g of *Cyathula prostrata* (Linn.) Blume per kilogram feed. Within rows, mean ± SEM with different superscript are significantly different at (*P* < 0.05) (*n* = 7).

## DISCUSSION

The problem of infertility in animals and man, in recent years, is receiving increasing research interest due to the decline in semen quality (Alrabeeah et al., 2014), and varied reasons are associated with male fertility problems. Research evidences have shown that more than 90% of the cases of male infertility are due to the problem of low sperm counts, poor sperm quality, or both (Bensdorp et al., 2007; Levine and Grifo, 2008). In this study, we noted that total sperm count was improved in a dose-related fashion at dosage levels of 10 and 20 g/kg feed than the dosage levels of 0 and 30 g/kg. This may be indicative of improved sperm production in buck rabbits following treatment with *C. prostrata* (Linn.) Blume. Previous studies have shown that some herbal plants can improve sperm production in animals and man (Mahdi et al., 2009; Anandakumar et al., 2013; Jaradat and Zaid, 2019). Increased total sperm count as noted in the present study has been associated with increased sperm production ability of the testis and increased transit time and the functionality of the epididymis (Creasy and Chapin, 2013). This may have resulted from enhanced testosterone and gonadotropins (FSH and LH) concentration, which stimulates spermatogenesis (Winters et al., 1979). In a similar experiment using a rat model, it was revealed that aqueous extract of *Rutacha lepensis* leaves at a dosage level of 0.5 to 2 g/kg significantly increased sperm count, motility, viability, and serum testosterone and FSH (Al Qarawi, 2005). In a recent study, we reported that *Moringa oleifera* Lam. leaf powder improves the semen volume, sperm count, and motility in a dose-dependent manner, with an increased concentration of FSH and LH in bucks (Ajuogu et al., 2019).

*Cyathula prostrata* (Linn.) Blume is used by ethnomedical and ethnoveterinary practitioners to treat several disease such as diabetes (Ogbonnia et al., 2016), cancer (Sowemimo et al., 2009), antibacterial and antifungi treatments (Oladimeji et al., 2005; Ogu et al., 2012), antioxidant, anti-inflammatory, and analgesic activities (Ibrahim et al., 2012), sores, articular rheumatism (Burkill, 1985), dysentery, wounds, and urethral discharges in many countries such as Nigeria, Cameron, Ghana, Ivory Coast, China, and Australia (Zheng and Xing, 2009; Alam et al., 2013). Several herbal plants are reported to improve male fertility potentials through the stimulation of healthy sperm production, improved libido/aphrodisiac properties, hormonal balance, and improved spermatozoa’s fertilizable properties in both animals and men (Nantia et al., 2009; Anandakumar et al., 2013; Jaradat and Zaid, 2019). Also, we previously reported that *M. oleifera* Lam. leaf powder enhances the male rabbit fertility potentials than in the females (Ajuogu et al., 2019).

However, the reduced sperm motility (asthenospermia) and sperm morphology (teratospermia) observed in this study may be related to the phytochemical content of the test plant that may have caused the reduced sperm quality (teratospermia and asthenospermia) (Kuete, 2014). The mechanism behind this is not clear; however, our study with *Custus afar* leaf on semen quality characteristics and serum testosterone revealed a similar trend, where the test plant significantly reduced sperm motility and morphology in buck rabbits (Ajuogu et al., 2018). Several other animal model studies have reported negative impact of some tropical herbal plants (Chinoy et al., 1995; Etta et al., 2012;Lampiao, 2013). For instance, Etta et al. (2012) observed a significant reduction in sperm count and motility and modification of sperm morphologies in rat treated with ethanol extract of *Phyllanthus amarus* Schum. et. Thonn.

## CONCLUSION

The increased sperm count observed in this study may represent an increased spermatogenesis in buck rabbits following treatment with *C. prostrata* (Linn.) Blume. However, the reduced sperm motility and morphology reported in our study may be related to spermatogenesis-associated problems traceable to the phyto content of the test plants. Therefore, more studies are recommended to further probe the impact of the phyto content of *C. prostrata* (Linn.) Blume on semen characteristics especially in relation to its role on poor sperm motility and morphology. Some tropical herbal plants and their extract inhibit male and female fertility and may be used to developed contraceptives for family planning in humans and *C. prostrata* (Linn.) Blume may be one of such plants that can be exploited for such use.
